# Supplementary material for: Resolving the full spectrum of human genome variation using Linked-Reads
Source: Genome Res. 2019 Apr;29(4):635–45. doi: 10.1101/gr.234443.118 (PMC6442396; doi:10.1101/gr.234443.118)
Supplement: Supplemental Material [file supp_gr.234443.118_Supplemental_longranger-2.2.zip › longranger-2.2.tar.bz2!/lr2.2-public/lib/stan/cmdstan/stan/doc/api/html/math_2functions_2is__uninitialized_8hpp_source.html]

Stan: src/stan/math/functions/is\_uninitialized.hpp Source File


|  |  |
| --- | --- |
|  | Stan  2.5.0  probability, sampling & optimization |


- Main Page
- Related Pages
- Namespaces
- Classes
- Files

- File List
- File Members

All Classes Namespaces Files Functions Variables Typedefs Enumerations Enumerator Friends Macros Pages

- src
- stan
- math
- functions

is\_uninitialized.hpp

Go to the documentation of this file.

1 #ifndef STAN\_\_MATH\_\_FUNCTIONS\_\_IS\_UNINITIALIZED\_HPP

2 #define STAN\_\_MATH\_\_FUNCTIONS\_\_IS\_UNINITIALIZED\_HPP

3

4 namespace stan {

5

6  namespace math {

7

18  template <typename T>

19  inline bool is\_uninitialized(T x) {

20  return false;

21  }

22

23  }

24 }

25 #endif

stan::math::is\_uninitialized

bool is\_uninitialized(T x)

Returns true if the specified variable is uninitialized.

**Definition:** is\_uninitialized.hpp:19


---

[ Stan Home Page ]

*© 2011–2014,
Stan Development Team.*
